# Cost-Effectiveness Analysis of Abemaciclib Plus Fulvestrant in the Second-Line Treatment of Women With HR+/HER2– Advanced or Metastatic Breast Cancer: A US Payer Perspective

**DOI:** 10.3389/fmed.2021.658747

**Published:** 2021-06-02

**Authors:** Yingcheng Wang, Mingjun Rui, Xin Guan, Yingdan Cao, Pingyu Chen

**Affiliations:** ^1^School of International Pharmaceutical Business, China Pharmaceutical University, Nanjing, China; ^2^Center for Pharmacoeconomics and Outcomes Research, China Pharmaceutical University, Nanjing, China

**Keywords:** cost-effectiveness, abemaciclib, CDK4/6 inhibitors, partitioned survival model, breast cancer

## Abstract

**Introduction:** This study evaluated the cost-effectiveness of abemaciclib plus fulvestrant (ABE + FUL) vs. palbociclib plus fulvestrant (PAL + FUL), ribociclib plus fulvestrant (RIB + FUL) and fulvestrant monotherapy (FUL) as second-line treatment for hormone receptor-positive and human epidermal growth factor receptor 2- negative advanced or metastatic breast cancer in the US.

**Methods:** The 3 health states partitioned survival (PS) model was used over the lifetime. Effectiveness and safety data were derived from the MONARCH 2 trial, MONALEESA-3 trial, and PALOMA-3 trial. Parametric survival models were used for four treatments to explore the long-term effect. Costs were derived from the pricing files of Medicare and Medicaid Services, and utility values were derived from published studies. Sensitivity analyses including one-way sensitivity analysis, probabilistic sensitivity analysis and scenario analysis were performed to observe model stability.

**Results:** In the PS model, compared with PAL + FUL, ABE + FUL yielded 0.44 additional QALYs at an additional cost of $100,696 for an incremental cost-utility ratio (ICUR) of $229,039/QALY. Compared with RIB + FUL, ABE + FUL yielded 0.03 additional QALYs at an additional cost of $518 for an ICUR of $19,314/QALY. Compared with FUL, ABE + FUL yielded 0.68 additional QALYs at an additional cost of $260,584 for ICUR of $381,450/QALY. From the PS model, the ICUR was $270,576 /QALY (ABE + FUL vs. PAL + FUL), dominated (ABE + FUL vs. RIB + FUL) and $404,493/QALY (ABE + FUL vs. FUL) in scenario analysis. In the probabilistic sensitivity analysis, the probabilities that ABE + FUL was cost-effective vs. PAL + FUL, RIB + FUL and FUL at thresholds of $50,000, $100,000, and $200,000 per QALY gained were 0% and the probabilities that ABE + FUL was cost-effective vs. PAL + FUL and RIB + FUL at thresholds of $50,000, $100,000, and $200,000 per QALY gained were 0.2, 0.6, and 7.3%.

**Conclusions:** The findings from the present analysis suggest that ABE + FUL might be cost-effective compared with RIB + FUL and not cost-effective compared with PAL + FUL and FUL for second-line treatment of patients with HR+/HER2– advanced or metastatic breast cancer in the US.

## Introduction

Breast cancer is the most common cancer among US women and the second major cause of cancer-related death (1). The global incidence and mortality rates for breast cancer were around 2.1 million (11.6%) and 627,000 (6.6%) in 2018, respectively (2). An estimated 250,000 new cases of breast cancer are diagnosed in the United States annually (3). The incidence of breast cancer in the US has reached 124 per 100,000 in 2015 (4). Nearly, 6% of patients are diagnosed with metastatic disease and approximately half of the patients with primary breast cancer will progress to the metastatic stage (5). A study reported the economic burden on breast cancer care for all women to be $16.5 billion in 2010 dollars. The substantial economic burden imposed by breast cancer in the United States is projected to increase to $20.5 billion by 2020 (6). According to Surveillance, Epidemiology, and End Results (SEER) registries, among patients with known hormone receptor (HR)/human epidermal growth factor receptor 2 (HER2) status in 2010, 36,810 (72.7%) were found to be HR+/HER2–, which is the most frequently diagnosed molecular subtype among all breast cancer types (7).

For hormone receptor-positive and human epidermal growth factor receptor 2- negative advanced or metastatic breast cancer (HR+/HER2-ABC/MBC), the mainstay of treatment is endocrine treatment (8). However, clinical resistance to endocrine therapy can be acquired by most patients, leading to disease progression (9). Therefore, cyclin-dependent kinase 4/6 inhibitors (CDK4/6i) in the setting of endocrine resistance have gained recent interest for improving the efficacy of existing therapies (5). CDK4/6i (palbociclib, ribociclib, and abemaciclib) in combination with fulvestrant have shown clinically meaningful efficacy and a good tolerability profile as second-line treatment in patients with HR+/HER2-ABC/MBC that progressed during prior endocrine therapy, within the PALOMA, MONALEESA, and MONARCH trials, respectively (10–15). Besides, fulvestrant+ CDK4/6 inhibitor is recommended as a preferred regimen for the second-line and subsequent therapy of HER2-negative ER- and/or PR-positive recurrent or stage IV breast cancer by NCCN guideline 2020. Additionally, some published meta-analyses confirmed the superiority of CDK4/6-inhibitor plus endocrine treatment over endocrine monotherapy based on progression-free survival and overall survival (16, 17). If this therapy is adopted in clinical, it will inevitably lead to an increase in the cost of medication.

Abemaciclib is a small-molecule inhibitor of CDK 4 and 6 that are involved in the cell cycle and promotion of cancer cell growth in case of unregulated activity, which was orally administered on a twice-daily continuous schedule (18). It is either given alone in patients who have undergone endocrine therapy and chemotherapy after the metastasis of cancer, or in combination with fulvestrant. Abemaciclib would induce G1 arrest and abrogate cell growth by preventing CDK 4 and 6 phosphorylation of the retinoblastoma tumor suppressor protein. Unlike other CDK inhibitors such as palbociclib and ribociclib, abemaciclib exhibits greater selectivity for CDK4 compared to CDK6 (19). On September 28, 2017, abemaciclib was approved in combination with fulvestrant for women with HR+/HER2-ABC/MBC with disease progression following endocrine therapy based on the MONARCH 2 trial, which was a randomized, multicenter, double-blind, placebo-controlled phase III trial (https://www.fda.gov/drugs/resources-information-approved-drugs/fda-approves-abemaciclib-hr-positive-her2-negative-breast-cancer).

MONARCH 2 trial assessed the efficacy of abemaciclib plus fulvestrant vs. placebo plus fulvestrant in patients with HR+/HER2-ABC that progressed during prior endocrine therapy. Abemaciclib plus fulvestrant significantly extended progression-free survival (PFS) median time when compared with placebo plus fulvestrant [16.4 vs. 9.3 months; hazard ratio (HR) 0.553; 95% CI, 0.449–0.681; *P* < 0.001] (14). A median overall survival (OS) was 46.7 months for abemaciclib plus fulvestrant and 37.3 months for placebo plus fulvestrant (HR 0.757; 95% CI, 0.606–0.945; *P* = 0.01) (15). Three published studies have assessed the cost-effectiveness of CDK4/6i plus fulvestrant as second-line therapy for HR+/HER2–ABC/MBC in the US, but no one focused on the cost-effectiveness of abemaciclib plus fulvestrant as second-line therapy. Therefore, the purpose of this article is to evaluate the cost-effectiveness of abemaciclib plus fulvestrant for the treatment of patients with HR+/HER2-ABC/MBC in the United States.

## Methods

### Model Overview

**Figure 1:**
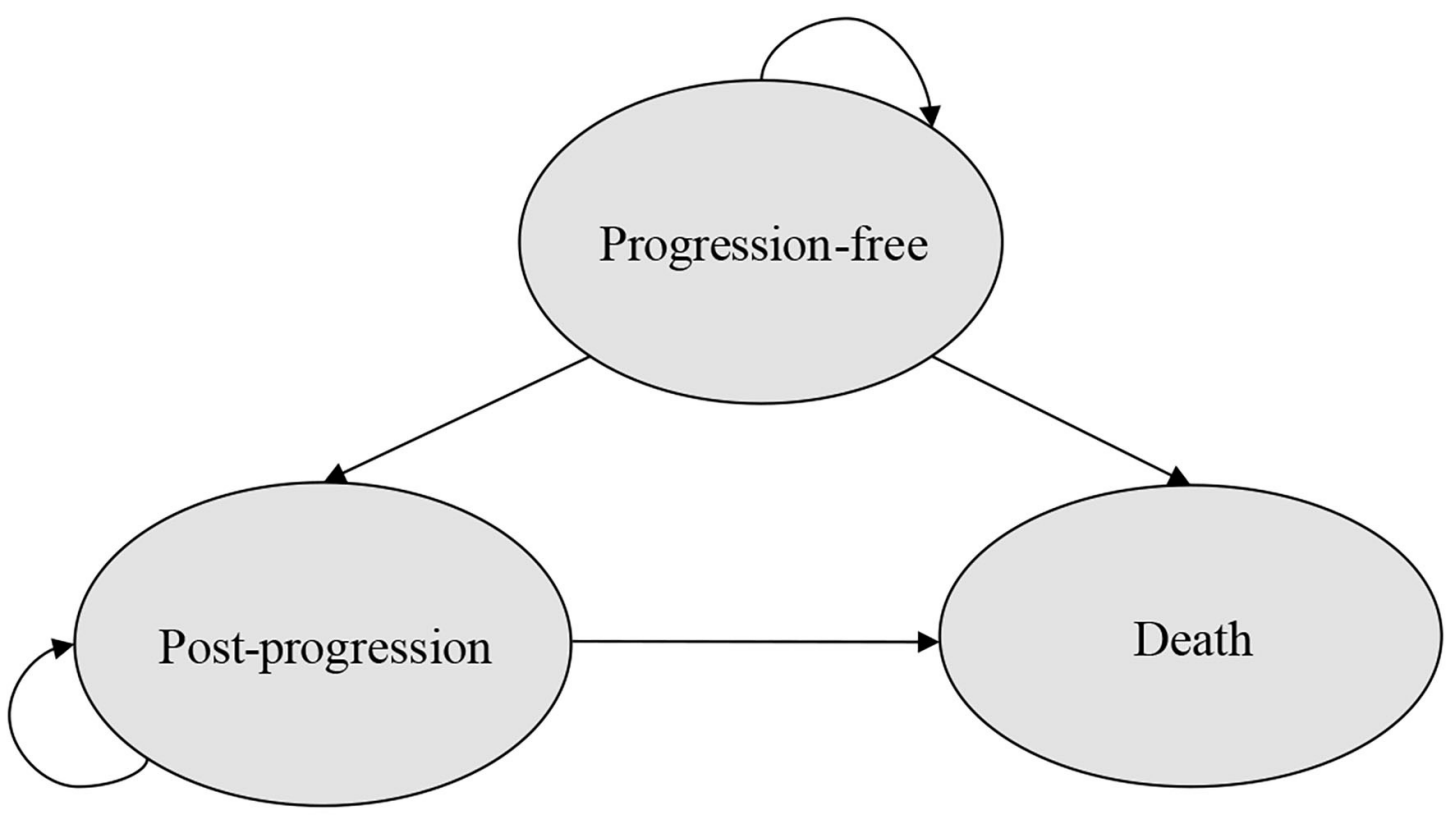
Structure of the PS model.

A partitioned survival model with three mutually exclusive health states (progression-free, post-progression, and death) was developed to estimate the costs and outcomes of patients with HR+/HER2-ABC/MBC that progressed during prior endocrine therapy in Microsoft Excel (Figure 1) from the perspective of US payer. For the partitioned survival (PS) model, progression-free survival (PFS) and overall survival (OS) curves were used to directly estimate the proportion of patients in each health state over time. The proportion of patients remaining in the PF state was directly provided by the PFS curve over time and the number of patients was derived by calculating the difference between the OS and the PFS curve at each time point in the post-progression (PP) state as this provided the proportion of patients who were alive but not progression-free (PF). Concerning the death state, the number of patients in the death state was calculated as 1 minus the OS curve at each time point (23).

The model assumed that all patients were in the PF health state at the beginning. Each cycle lasted 4 weeks, which was consistent with the dose schedule in MONARCH 2 trial (14, 15), MONALEESA-3 trial (12, 13) and PALOMA-3 trial (10, 11). A lifetime horizon was chosen, and modeled until ≥95% of patients in all groups died. The following outcomes were examined: quality-adjusted life-years (QALYs), life-years gained (LYGs), costs, and all of them were discounted by 3%.

### Cohort Patients

The target population in the model is those women with HR+/HER2-ABC/MBC who had progressed while receiving previous endocrine therapy. The intervention group receives abemaciclib 150 mg twice daily during each 28-days cycle combined with 500 mg fulvestrant by intramuscular injection on days 1 and 15 of the first cycle, and day 1 of subsequent cycles. Three control groups were set up to compare the cost-effectiveness between abemaciclib combined with fulvestrant treatment (ABE + FUL) and other CDK4/6 inhibitors (palbociclib and ribociclib) combined with fulvestrant treatment and fulvestrant monotherapy.

Control group 1 (PAL + FUL): In group 1, patients received 125 mg palbociclib once daily for 3 weeks, followed by a week off in one cycle, and 500 mg fulvestrant by intramuscular injection on days 1 and 15 of the first cycle, and day 1 of subsequent cycles.

Control group 2 (RIB + FUL): In group 2, patients received 600 mg ribociclib per day for 3 weeks followed by a week off and 500 mg fulvestrant by intramuscular injection on days 1 and 15 of the first cycle, and day 1 of subsequent cycles.

Control group 3 (FUL): Patients only received 500 mg fulvestrant by intramuscular injection on days 1 and 15 of the first cycle, and day 1 of subsequent cycles.

All patients received the treatment until the disease progressed. After progression, patients would receive subsequent treatments, which were composed of anthracyclines, taxanes, anti-metabolites, vinca alkaloids, hormones, HER2-targeted therapies and non-HER2-targeted therapies. The proportions of these therapies were derived from the study of Sorensen et al. (24).

### Clinical Efficacy

The clinical efficacy parameters in the model were mainly derived from three clinical trials (MONARCH 2, PALOMA-3, and MONALEESA-3). PALOMA-3 trial and MONALEESA-3 trial, respectively, compared the effects and safety of palbociclib and ribociclib combined with fulvestrant and fulvestrant monotherapy in patients with HR+/HER2-ABC. They both showed significant advantages compared with fulvestrant monotherapy. The detailed information of three clinical trials has been listed in the Supplementary Materials.

The effects of these four treatment options were indirectly compared with the fulvestrant monotherapy group as control. Extrapolation was required in the cost-effectiveness analysis for the limited follow-up time of the K-M curves in clinical trials. The standard parametric models were fitted using Exponential, Weibull, Gompertz, Log-logistic, Log-normal distributions, which determined by Akaike information criterion (AIC) and the Bayesian information criterion (BIC) goodness-of-fit statistics and visual inspection. For PAL + FUL and FUL, the standard parametric models were separately used to extrapolate the PFS and OS curves derived from PALOMA-3 trial, because they did not meet the proportional hazard (PH) assumption. For RIB + FUL and ABE + FUL, the HR of PFS and OS from the MONARCH-2 trial and MONALEESA-3 trial were used since they almost met the proportional hazard assumption.

**Figure 2:**
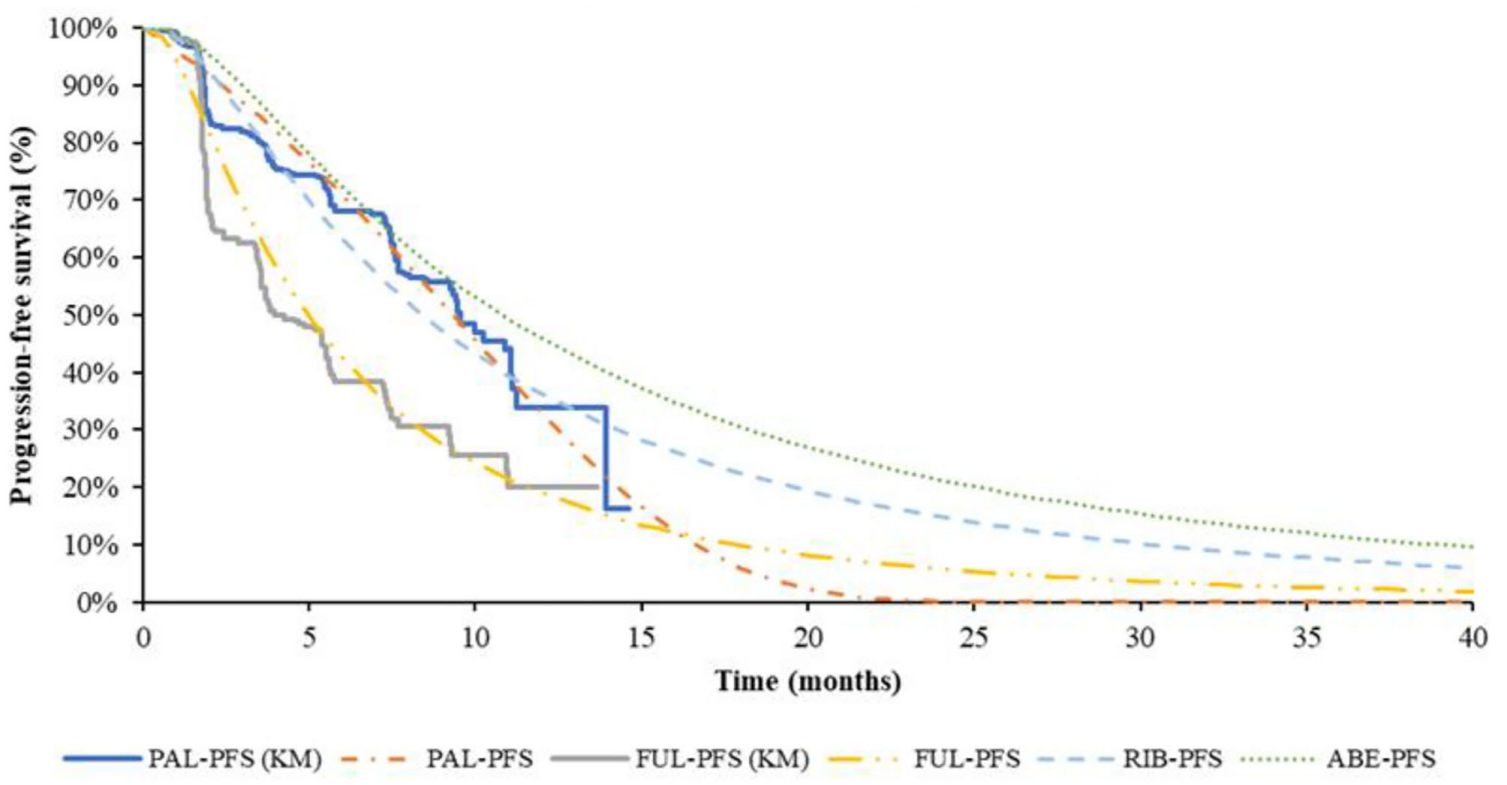
K-M curve and parametric survival distributions for PFS. PFS, progression-free survival; ABE, abemaciclib; RIB, ribociclib; PAL, palbociclib; FUL, fulvestrant.

**Figure 3:**
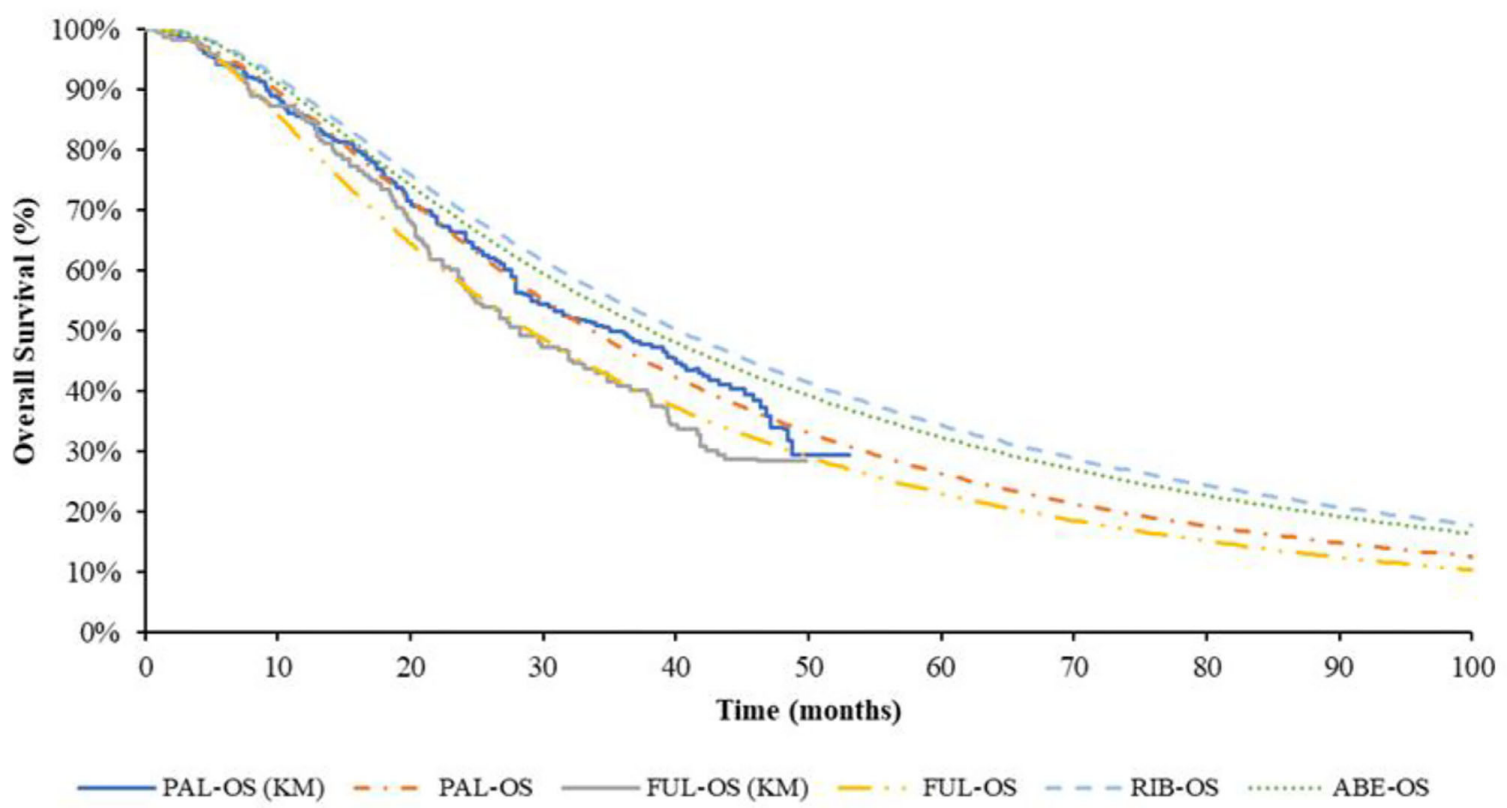
K-M curve and parametric survival distributions for OS. OS, overall survival; ABE, abemaciclib; RIB, ribociclib; PAL, palbociclib; FUL, fulvestrant.

The pseudo-individual patient data (IPD) was extracted with Engauge Digitizer software from the PALOMA-3 trial, reconstructed through R 3.6.0 (25) and fitted by the standard parametric models through R 3.6.0. For PFS of fulvestrant monotherapy group, the preferred distribution for the base case was Log-normal, which demonstrated the best fit to survival based on AIC and BIC. The Log-logistic model was the preferred distribution for OS of fulvestrant monotherapy group, but for the median value of suboptimal Lognormal distribution was closer to real value. Log-normal distribution was selected in the base-case model finally. For PFS of PAL + FUL, the preferred distribution for the base case was Gompertz distribution. According to the results of AIC and BIC, the Weibull distribution was the best fitting distribution of the OS curve of PAL + FUL. Considering Weibull distribution would lead to logic errors after extrapolation, the suboptimal distribution Log-logistic was selected finally. The reconstructed K-M curve of PFS and OS, as well as the fitted curve, were shown in Figures 2, 3, respectively. The values of AIC and BIC could also be found in the Supplementary Materials.

The study of Tevaarwerk et al. (26) showing the 15-year survival probability of advanced breast cancer patients was ~5%. In this study, when 95% of the patients died, the time for every group was ABE + FUL: 15.67 years; RIB + FUL: 16.5 years, PAL + FUL: 14.75 years and FUL: 11.9 years, which is consistent with the long-term survival in the above study.

### Cost

All costs were in 2019 US dollars inflated using the Consumer Price Index (https://fred.stlouisfed.org/series/CPIMEDSL). Only the direct medical care costs were included as the US payer perspective was taken. These included drug acquisition, administration, and medical costs associated with each state, end of life care costs, and AE costs. All costs were derived from database data (such as Centers for Medicare and Medicaid Services) or published literature. The drug unit costs were derived from Centers for Medicare and Medicaid Services (such as Medicare Part B and Medicare Part D), and the costs in each cycle were calculated. The drug costs for subsequent treatment came from published literature. Since fulvestrant monotherapy was administrated in injection, the drug administration costs were included.

When patients were in the PF state, they needed to be followed up and monitored until the disease progressed, which mainly included lab scans and tests as well as bone metastases management. Costs of patients in PP state included the cost of subsequent treatment drugs and best supportive care costs. The proportion of patients who received subsequent treatments was obtained from the results of related clinical trials (11, 13, 15), and the costs of subsequent treatments were calculated by multiplying the cost in each cycle by the number of cycles. The impact of grade 3 or 4 AEs (≥5%) was considered in the model, and related costs were derived from the published literature. Table 1 provides an overview of all costs and AE probabilities.

**Table 1:** Overview of all model parameters.

**Model inputs**	**Value**	**Distribution**	**Low**	**High**	**Source**
HR PFS of ribociclib	0.59	CONSTANT	/	/	(12)
HR PFS of abemaciclib	0.46	CONSTANT	/	/	(14)
HR OS of ribociclib	0.72	CONSTANT	/	/	(13)
HR OS of abemaciclib	0.76	CONSTANT	/	/	(15)
**Direct costs per cycle**					
Palbociclib	$11,938	CONSTANT	$9,550	$11,938	Medicare Part D
Ribociclib	$13,110	CONSTANT	$10,488	$13,110	Medicare Part D
Abemaciclib	$11,815	CONSTANT	$9,452	$11,815	Medicare Part D
Fulvestrant	$1,808	CONSTANT	$1,446	$1,808	Medicare Part B
Medical follow-up	$2,959	GAMMA	$2,367	$3,551	(24)
Drug administration	$702	GAMMA	$561	$842	(27)
After progression	$6,549	GAMMA	$5,240	$7,859	(24)
Subsequent treatment	$9,061	GAMMA	$7,248	$10,873	(24)
End of life care	$2,601	GAMMA	$2,081	$3,121	(24)
**Costs of MAEs per event**					
Neutropenia	$16,256	GAMMA	$14,871	$17,642	(28)
Leucopenia^*^	$16,256	GAMMA	$14,871	$17,642	(28)
Diarrhea	$11,545	GAMMA	$9,170	$13,920	(28)
Anemia	$14,532	GAMMA	$13,262	$15,804	(28)
Infections	$14,595	GAMMA	$11,248	$18,425	(28)
Hepatobiliary toxicity	$7,516	GAMMA	$6,013	$9,019	(27)
**Proportion of patients received subsequent treatment**					
Palbociclib	71%	BETA	64%	96%	(11)
Ribociclib	61%	BETA	57%	85%	(13)
Abemaciclib	63%	BETA	49%	73%	(15)
Fulvestrant	80%	BETA	50%	76%	(11)
**Risk of AEs in palbociclib**					
Neutropenia	0.696	BETA	0.557	0.835	(11)
Leucopenia	0.383	BETA	0.306	0.460	(11)
Infections	0.052	BETA	0.042	0.062	(11)
Anemia	0.043	BETA	0.034	0.052	(11)
**Risk of AEs in ribociclib**					
Neutropenia	0.571	BETA	0.457	0.685	(13)
Leucopenia	0.155	BETA	0.124	0.186	(13)
Hepatobiliary toxicity	0.137	BETA	0.110	0.164	(13)
Infections	0.077	BETA	0.062	0.092	(13)
Anemia	0.039	BETA	0.031	0.047	(13)
**Risk of AEs in abemaciclib**					
Neutropenia	0.297	BETA	0.238	0.356	(15)
Leucopenia	0.111	BETA	0.089	0.133	(15)
Diarrhea	0.145	BETA	0.116	0.174	(15)
Anemia	0.090	BETA	0.072	0.108	(15)
**Risk of AEs in fulvestrant**					
Neutropenia	0.006	BETA	0.005	0.007	(11)
Leucopenia	0.035	BETA	0.028	0.042	(11)
Anemia	0.023	BETA	0.018	0.028	(11)
**QoL utility (per year)**					
PF	0.837	BETA	0.753	0.921	(29)
PP	0.443	BETA	0.399	0.487	(30)
**Disutilities of AEs (per year)**					
Neutropenia	−0.2466	CONSTANT	−0.2713	−0.2220	(31)
Leucopenia^*^	−0.2466	CONSTANT	−0.2713	−0.2220	(31)
Diarrhea	−0.1198	CONSTANT	−0.1318	−0.1078	(31)
Anemia	−0.1914	CONSTANT	−0.2105	−0.1722	(31)
Infections	−0.2303	CONSTANT	−0.2534	−0.2073	(31)
Hepatobiliary toxicity	−0.3080	CONSTANT	−0.3696	−0.2464	(32)
**AEs duration (days)**					
Neutropenia	2	CONSTANT	/	/	(33)
Leucopenia^*^	2	CONSTANT	/	/	(33)
Diarrhea	2	Normal	/	/	(34)
Anemia	21	Normal	/	/	(33)
Infections	4.2	Normal	/	/	(35)
Hepatobiliary toxicity	4.3	Normal	/	/	(35)
Duration of subsequent treatment (cycles)	4.66	Normal	3.73	5.59	(36)
Discount rate	3%	CONSTANT	0%	5%	(29)

*PFS, progression-free survival; OS, overall survival; HR, hazard ratio; MAE, main adverse event*.

* *The parameters of leucopenia were assumed to be the same as those of neutropenia*.

*Web of Medicare Part D: https://portal.cms.gov/wps/portal/unauthportal/unauthmicrostrategyreportslink?evt=2048001&src=mstrWeb.2048001&documentID=203D830811E7EBD800000080EF356F31&visMode=0*.

*Web of Medicare Part B: https://portal.cms.gov/wps/portal/unauthportal/unauthmicrostrategyreportslink?evt=2048001&src=mstrWeb.2048001&documentID=AEC7511A11E817EF2FBA0080EFC5E3D8&visMode=0&currentViewMedia=1&Server=E48V126P&Project=OIPDABI_Prod&Port=0&connmode=8&ru=1&share=1&hiddensections=header,path,dockTop,dockLeft,footer*.

### Utility

Utility values for PF were derived from the study of Mistry et al. (29). This utility was calculated using the latest UK value set and data was from EuroQol 5-dimension 5-level (EQ-5D-5L) data collected in the MONALEESA-2 trial. Utility value for PD was derived from the study reported by Lloyd et al. (30), which reported estimated health-state utility values by using the standard gamble technique. Disutilities for adverse events (AEs) whose values were taken from published sources were included. The duration of AEs was derived from the published clinical expert opinion. Table 1 showed the utility values applied to the PF and PP health states, the disutility values associated with AEs and mean AE duration.

### Sensitivity Analyses

One-way sensitivity analysis was performed to identify the parameters to which the model was most sensitive. The upper and lower limits of the inputs were defined by 95% confidence intervals where possible and derived from the original literature or with plausible variation around the base-case values by 10% in utilities and 20% in other parameters. Probabilistic sensitivity analysis (PSA) was conducted using 1,000 iterations to examine parameter uncertainty over the entire model, and cost-effectiveness acceptability curves were then calculated. Distributions of the parameters in the model were mainly referred to the model book (37) and the published cost-effectiveness analysis paper (33). Additionally, all parameters in the parametric survival model were assessed through Cholesky decomposition.

For the inconsistent source of the utilities used in the base-case analysis, the utilities reported by Beauchemin et al. (31) which were obtained from an exhaustive review of the literature were selected for the scenario analysis (PF utility = 0.76, PP utility = 0.55).

### Ethics

The data of the PFS/OS curves were based on previously published trials, not from the database or the medical records. We used the Engauge Digitizer software to get the survival information from the figures in the paper and reconstructed data by ourselves, which was explained in the section Clinical Efficacy. Besides, the utilities and costs were derived from the published literature, so ethics approval or specific consent procedures were not required for this study.

## Results

### Base-case Results

**Figure 4:**
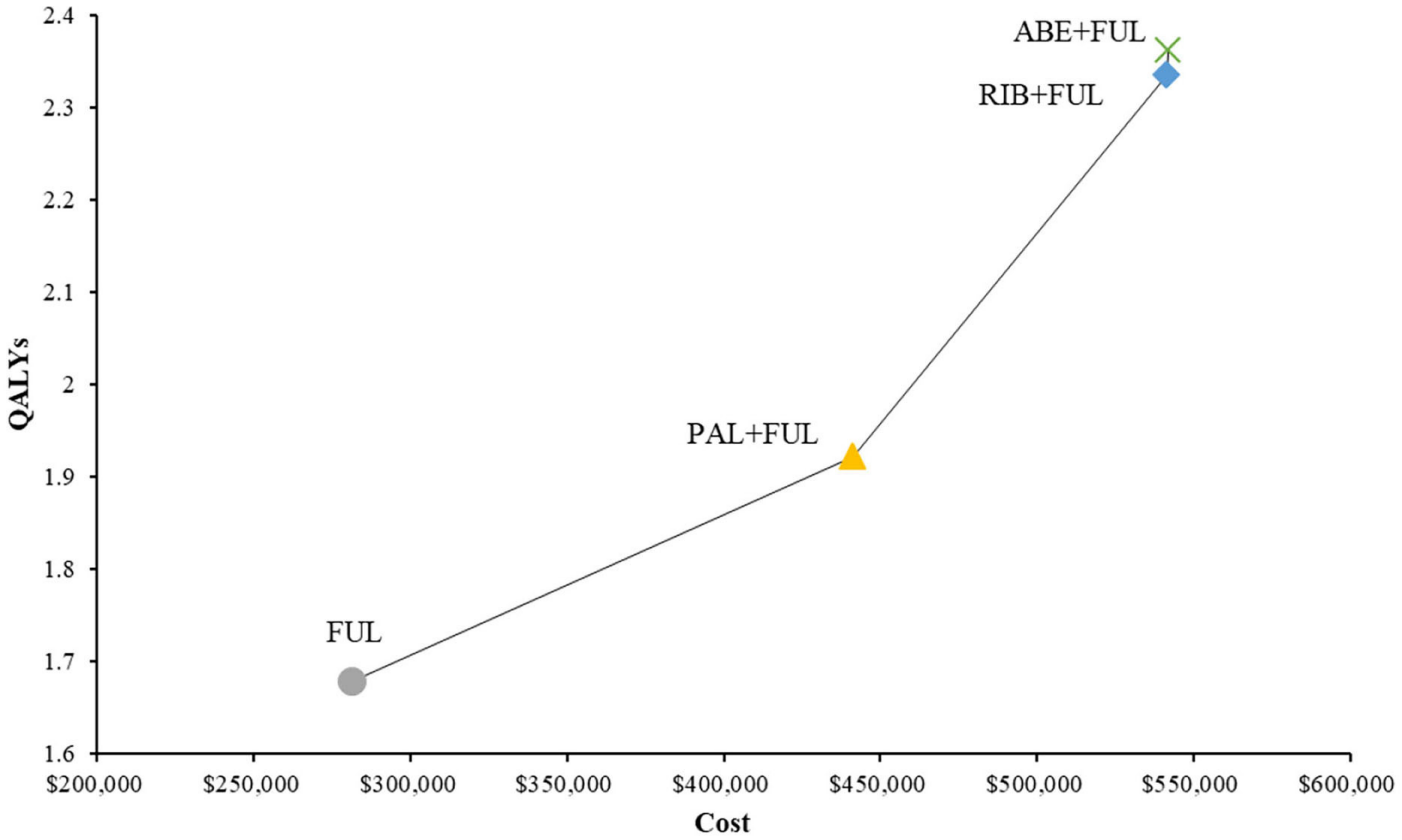
Cost-effectiveness plane of different treatment options. QALYs, quality-adjusted life years; ABE, abemaciclib; RIB, ribociclib; PAL, palbociclib; FUL, fulvestrant.

ABE + FUL had a total cost of $541,890 vs. 441,194 for PAL + FUL, $541,372 for RIB + FUL and $281,306 for FUL (Table 2). Total LYs for each treatment option were 4.09 for ABE + FUL, 3.65 for PAL + FUL, 4.29 for RIB + FUL and 3.20 for FUL. Total QALYs for each treatment were 2.36 for ABE + FUL, 1.92 for PAL + FUL, 2.33 for RIB + FUL and 1.68 for FUL. In order to compare four treatments directly, all results were shown in the cost-effectiveness plane (Figure 4).

**Table 2:** Base-case results.

**Outcomes**	**ABE + FUL**	**RIB + FUL**	**PAL + FUL**	**FUL**
LYs	4.09	4.29	3.65	3.20
QALYs	2.36	2.33	1.92	1.68
Cost, $	$541,890	$541,372	$441,194	$281,306
Incremental cost	/	$518	$100,696	$260,584
Incremental QALYs	/	0.03	0.44	0.68
Incremental cost per LY, $	/	Dominated	$229,689	$291,323
Incremental cost per QALY, $	/	$19,314	$229,039	$381,450

*LYs, life years; QALYs, quality-adjusted life years; ABE, abemaciclib; RIB, ribociclib; PAL, palbociclib; FUL, fulvestrant*.

In terms of incremental costs and QALYs, ABE + FUL was associated with the additional cost of $100,696 and a gain of 0.44 QALYs compared with PAL + FUL, giving an ICUR of $229,039 per QALY gained (Table 2). Compared with RIB + FUL, ABE + FUL was associated with an incremental cost of $518 and an incremental QALY gain of 0.03, giving an ICUR of $19,314 per QALY gained. Compared with FUL, ABE + FUL was associated with an incremental cost of $260,584 and an incremental QALY gain of 0.68, giving an ICUR of $381,450 per QALY gained. Table 2 shows the base-case results in the model.

### Sensitivity Analyses

**Figure 5:**
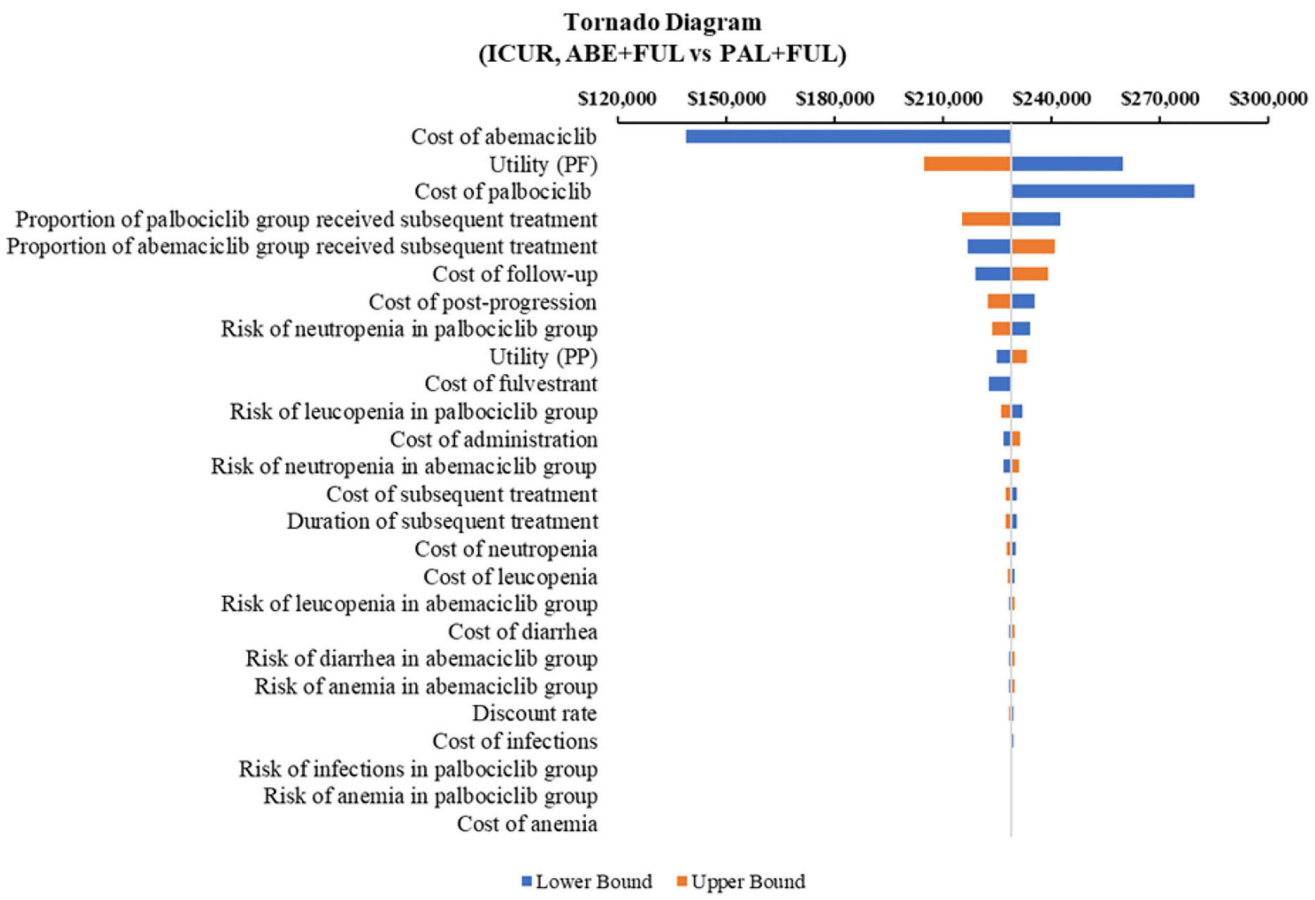
Tornado diagram of the abemaciclib plus fulvestrant vs. palbociclib plus fulvestrant. ICUR, incremental cost-utility ratio; ABE, abemaciclib; PAL, palbociclib; FUL, fulvestrant.

**Figure 6:**
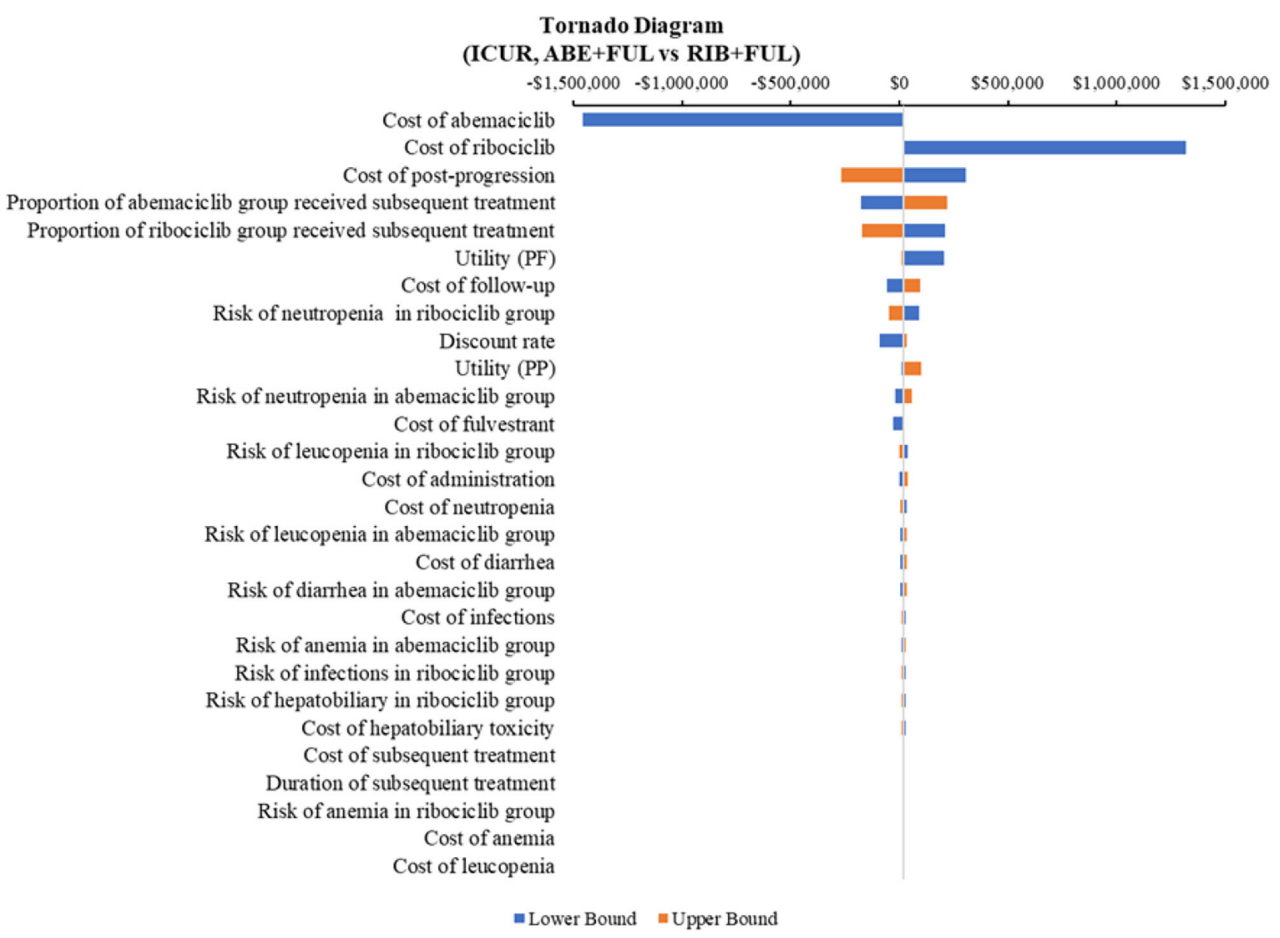
Tornado diagram of the abemaciclib plus fulvestrant vs. ribociclib plus fulvestrant. ICUR, incremental cost-utility ratio; ABE, abemaciclib; RIB, ribociclib; FUL, fulvestrant.

**Figure 7:**
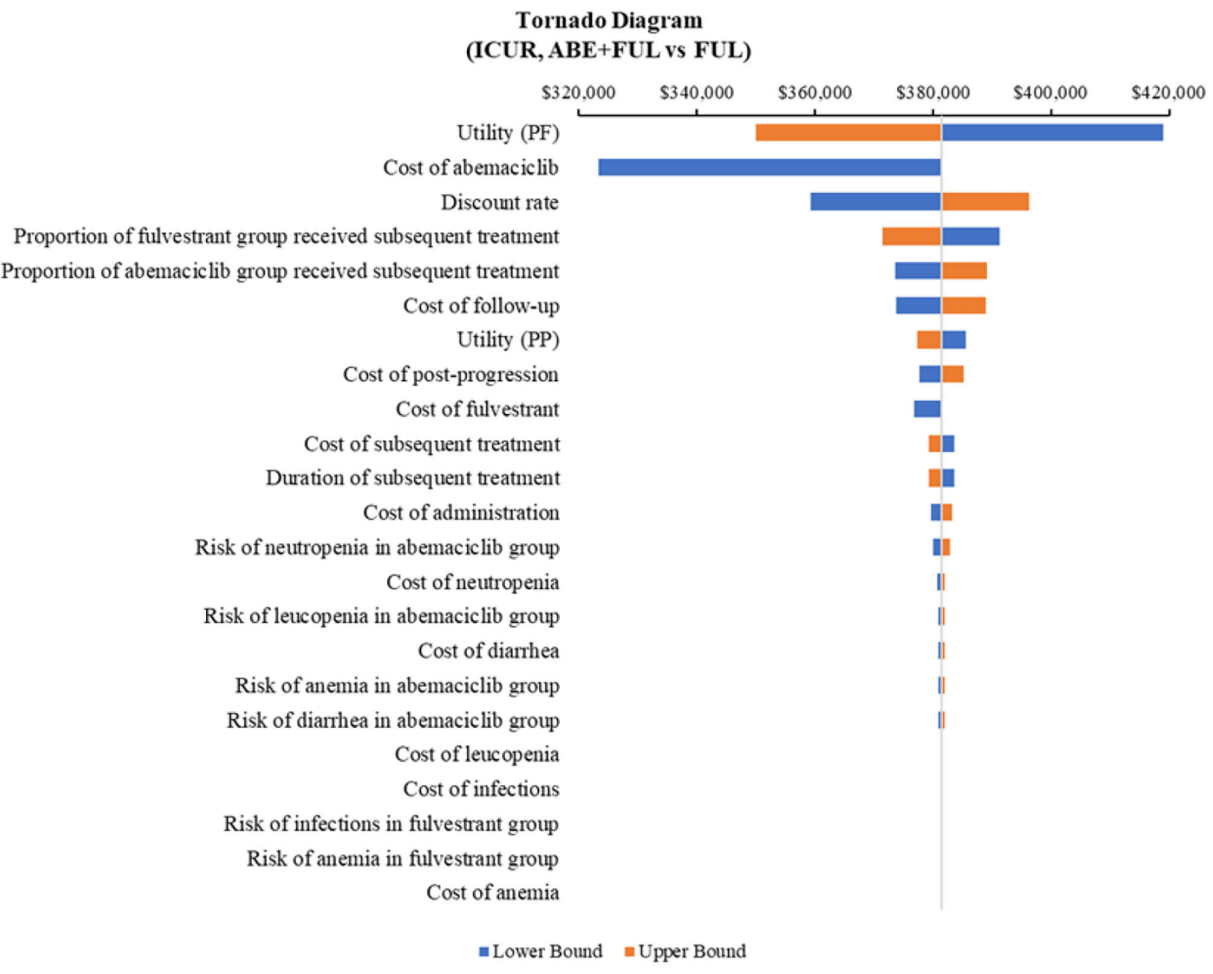
Tornado diagram of the abemaciclib plus fulvestrant vs. fulvestrant monotherapy. ICUR, incremental cost-utility ratio; ABE, abemaciclib; FUL, fulvestrant.

According to the DSA, for ABE + FUL vs. PAL + FUL, the key model drivers were cost of abemaciclib, utility (PF) and cost of palbociclib (Figure 5). Compared with RIB + FUL, the key model drivers were cost of abemaciclib, cost of ribociclib and cost of post-progression (Figure 6). Compared with FUL, the key model drivers were cost of abemaciclib, discount rate and proportion of fulvestrant group received subsequent treatment (Figure 7).

**Figure 8:**
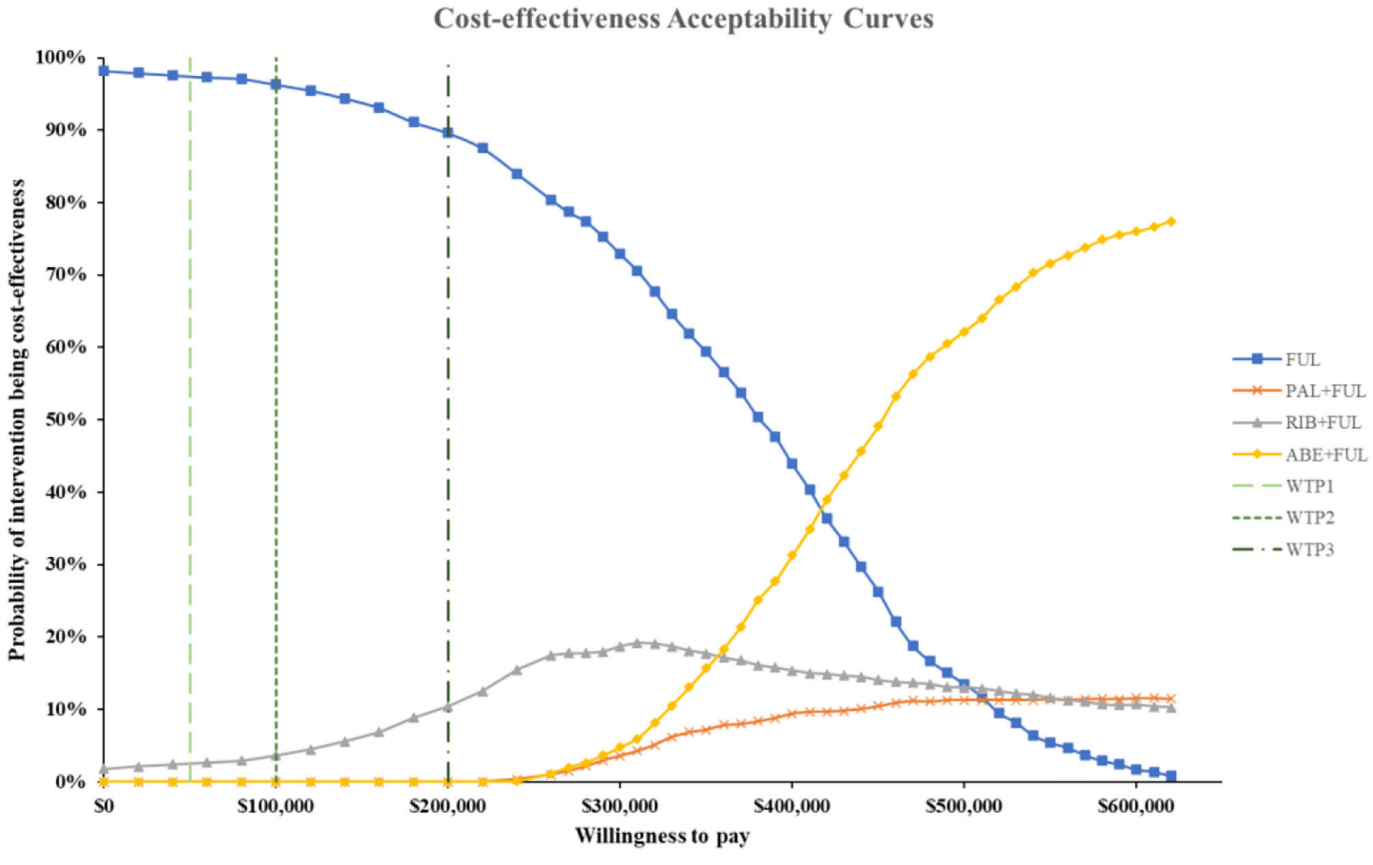
Cost-effectiveness acceptability curves for abemaciclib plus fulvestrant vs. palbociclib plus fulvestrant, ribociclib plus fulvestrant and fulvestrant monotherapy. ABE, abemaciclib; RIB, ribociclib; PAL, palbociclib; FUL, fulvestrant; WTP1, $50,000; WTP2, $100,000; WTP3, $200,000.

**Figure 9:**
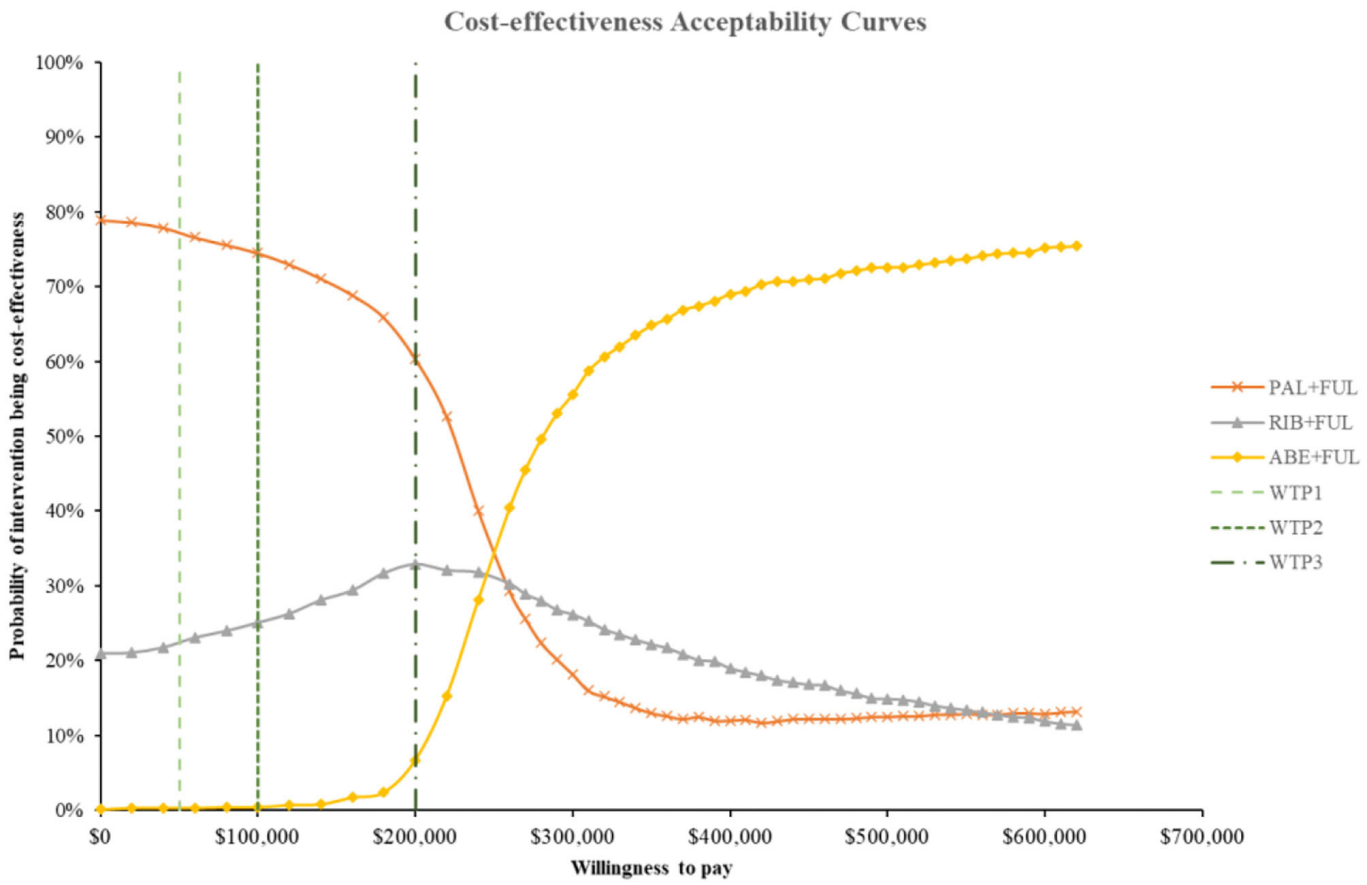
Cost-effectiveness acceptability curves for abemaciclib plus fulvestrant vs. palbociclib plus fulvestrant, ribociclib plus fulvestrant. ABE, abemaciclib; RIB, ribociclib; PAL, palbociclib; FUL, fulvestrant; WTP1, $50,000; WTP2, $100,000; WTP3, $200,000.

According to the PSA, ABE + FUL was associated with a mean incremental cost increase of $102,001, 899, and 261,175 and a main increase of 0.45, 0.03, and 0.69 QALYs compared with PAL + FUL, RIB + FUL and FUL, giving an ICUR of $ 227,471, 19,314, and 379,754 per QALY gained. The probabilities that ABE + FUL was cost-effective vs. PAL + FUL, RIB + FUL and FUL at thresholds of $50,000, 100,000, and 200,000 per QALY gained were 0%, and the cost-effectiveness acceptability curves among the four treatments options were presented in Figure 8. The probabilities that ABE + FUL was cost-effective vs. PAL + FUL and RIB + FUL at thresholds of $50,000, 100,000, and 200,000 per QALY gained were 0.2, 0.6, and 7.3%. The cost-effectiveness acceptability curves for them were presented in Figure 9.

According to the scenario analysis where the PF utility of 0.76 and the PP utility of 0.55 were used, the result showed that the ICUR was $270,576/QALY (ABE + FUL vs. PAL + FUL), dominated (ABE + FUL vs. RIB + FUL) and $404,493/QALY (ABE + FUL vs. FUL).

## Discussion

For patients with HR+/HER2-ABC who had developed endocrine resistance, CDK4/6 inhibitors added to/combined with endocrine therapy provide a new treatment option instead of cytotoxic chemotherapy. In this study, the cost-effectiveness of abemacicilb was estimated in combination with fulvestrant in the second-line treatment of women with HR+/HER2– ABC/MBC. To the best knowledge, this is the first study to explore the cost-effectiveness of abemaciclib, and also the first study to compare the cost-effectiveness of three approved CDK4/6 inhibitors in the US The results of this study revealed the difference between the long-term clinical effects and costs of four treatment options, suggesting fulvestrant monotherapy would be the most cost-effective treatment among these four choices under the US willingness to pay. If only three CDK4/6 inhibitors were compared, PAL + FUL has the highest probability to be cost-effective.

In the United States, traditionally accepted thresholds for cost-effectiveness ratio are $50,000 per QALY. Since some evidence suggests that this was too low, the study used the recommended thresholds of $50,000, 100,000, and 200,000 per QALY (38). In the base case analysis, the ABE + FUL was cost-effective only when compared to RIB + FUL, resulting in an ICUR of $19,314/QALYs. The outcomes between them were close. For ABE + FUL, it gained additional 0.03 QALYs, while RIB + FUL brought more LYs, and the incremental cost was only $518. This result was caused by the longer PFS of ABE + FUL group but the similar OS between them. Thus, ABE + FUL spent more on drug costs and led to the higher quality of life. However, a recent network meta-analysis found no significant differences among the three CDK4/6 inhibitors in combination with fulvestrant in terms of PFS (17). Based on this clinical efficacy, the cost-minimization analysis (CMA) might be more suitable to access the cost-effectiveness between these three treatment options. The result was also sensitive to changes in utility value, as shown in the scenario analysis. RIB + FUL dominated ABE + FUL when using utility values from another source. Since the gap between these two treatments is small, a little change in model inputs can cause extremely different results. Therefore, the cost-effectiveness result between these two options was not robust, which needed more reliable data sources. Different from the other two CDK4/6 inhibitors, abemaciclib has also been approved by FDA as monotherapy for the treatment of adult patients with HR+/HER2- ABC/MBC with disease progression following endocrine therapy and prior chemotherapy (https://www.fda.gov/drugs/resources-information-approved-drugs/fda-approves-abemaciclib-hr-positive-her2-negative-breast-cancer). Although the cost-effectiveness analysis of abemaciclib monotherapy was not evaluated in this study, it may have potential advantages in cost-effectiveness.

When compared to the PAL + FUL, ABE + FUL resulted in high costs and also improved LYs as well as QALYs, giving an ICER of $229,689/LYs and an ICUR of $229,039/QALYs. The results were robust in sensitivity analyses, while only the cost of abemaciclib and palbociclib play an important role. ABE + FUL has better clinical efficacy and fewer adverse events than PAL + FUL, but the probability of being cost-effective than PAL + FUL was still relatively low even under the WTP of $200,000.

When compared to fulvestrant monotherapy, ABE + FUL generated an ICUR of $381,450/QALYs which exceeded the US threshold. Some published studies have already evaluated the cost-effectiveness of PAL + FUL and RIB + FUL with fulvestrant monotherapy in the US (20–22). These studies had reached a common conclusion that when CDK4/6 inhibitors were added to endocrine therapy, it was not a cost-effective choice at current drug prices in the United States. In summary, existing evidence showed the base case result was unstable, and if their effects were assumed to be the same, the CMA might be more suitable. It is recommended to combine the results of PSA and scenario analyses as references for decision-making.

This analysis also had several limitations. First, for no head-to-head clinical trials were available and the lack of IPD, only the indirect comparison was applied into the model to compare the cost-effectiveness of ABE + FUL with the other three treatment options, so there might exist certain deviations in the indirect comparison. The comparable baseline population characteristics in three clinical trials (MONARCH 2, PALOMA-3, and MONALEESA-3) have been listed and compared, and they seemed similar. Galve-Calvo et al. used matching-adjusted indirect comparison (MAIC) to match the individual data of the MONALEESA-2 study with PALOMA-1 and PALOMA-2 studies to obtain the adjusted HR of ribociclib plus letrozole vs. letrozole monotherapy in order to evaluate the cost-effectiveness between ribociclib plus letrozole and palbociclib plus letrozole in the first-line treatment of HR+/HER2- ABC patients in Spain. According to the results from the study, there was little difference between the adjusted HR and non-adjusted HR if the baseline population characteristics were similar in trials (39). Second, appropriate utility of target patients based on the American population was not available, so the utility input in this model was not from the same source and the same situation also occurred in the study of Mistry et al. (29). Therefore, scenario analysis was conducted to explore the uncertainty of health state utility from different sources and the result had been discussed. Third, long-term survival data of HR+/HER2-ABC patients using CDK4/6 inhibitors had not been found to validate the reliability of the extrapolated results of PFS and OS. Though compared with the study of Tevaarwerk et al. which only given the survival probability for the HR+ MBC patients (26), the specific extrapolated PFS or OS of each treatment option still cannot be validated precisely. However, although survival probability was different between treatment options, the overall results were similar and close in the above study.

## Conclusion

Based on the results of this cost-effectiveness analysis and sensitivity analyses, second-line treatment of women with HR+/HER2-ABC/MBC with abemaciclib plus fulvestrant may generate QALY gains and cost increase compared with ribociclib plus fulvestrant therapy and be cost-effective at current willingness-to-pay thresholds in the United State. Besides, abemaciclib plus fulvestrant was not cost-effective compared with palbociclib plus fulvestrant and fulvestrant monotherapy for second-line treatment of patients with HR+/HER2- ABC/MBC in the US.

## Data Availability Statement

The original contributions presented in the study are included in the article/Supplementary Material, further inquiries can be directed to the corresponding author/s.

## Ethics Statement

The data of the PFS/OS curve was based on previously published trial, not from the database or the medical records. We used the Engauge Digitizer software to get the survival information from the figures in the paper and reconstructed data by ourselves, which was explained in the section Clinical Efficacy. Besides, the utilities and costs were derived from the published literatures, so ethics approval or specific consent procedures were not required for this study.

## Author Contributions

YW, MR, XG, and PC developed the economic model and performed the analyses. YW, MR, and YC interpreted the results and wrote the draft manuscript. MR, YW, YC, and PC reviewed, analyzed, and interpreted the data. XG and YW contributed to the design of the primary model and the interpretation of the results. All authors reviewed and approved the final version.

## Conflict of Interest

The authors declare that the research was conducted in the absence of any commercial or financial relationships that could be construed as a potential conflict of interest.
