# Supplementary material for: Cost-Effectiveness Analysis of Abemaciclib Plus Fulvestrant in the Second-Line Treatment of Women With HR+/HER2– Advanced or Metastatic Breast Cancer: A US Payer Perspective
Source: Front Med (Lausanne). 2021 Jun 2;8:658747. doi: 10.3389/fmed.2021.658747 (PMC8206485; doi:10.3389/fmed.2021.658747)
Supplement: Supplementary file 1 [file Data_Sheet_1.docx]

# **Supplementary Material**

Cost-Effectiveness analysis of Abemaciclib plus Fulvestrant in the Second-Line Treatment of Women with HR+/HER2- Advanced or Metastatic Breast Cancer: A U.S. Payer Perspective

**Table S1** Results of fitting to the observed data

**Table S2** Best fitting and the value of the parameter

**Table S3** Detailed information of MONARCH 2

**Table S4** Detailed information of MONALEESA-3

**Table S5** Detailed information of PALOMA-3

**Table S6** Efficacy results of three clinical trials

**Table S7** Comparable baseline population characteristics in three clinical trials

# Estimation of Parametric Survival Distributions for Overall Survival and Progression-Free Survival

Table S1 Results of fitting to the observed data

|  |  | Exponential | Weibull | Gompertz | Log-normal | Log-logistic |
| --- | --- | --- | --- | --- | --- | --- |
| PFS |  |  |  |  |  |  |
| FUL | AIC | 509.77 | 509.29 | 508.21 | 474.40 | 482.08 |
|  | BIC | 516.09 | 512.45 | 514.53 | 480.72 | 488.40 |
| PAL+FUL | AIC | 904.72 | 841.06 | 821.88 | 888.62 | 885.67 |
|  | BIC | 908.57 | 848.76 | 829.58 | 896.32 | 893.37 |
| OS |  |  |  |  |  |  |
| FUL | AIC | 457.85 | 445.29 | 453.91 | 444.68 | 439.97 |
|  | BIC | 461.01 | 451.61 | 460.23 | 451.00 | 446.29 |
| PAL+FUL | AIC | 900.75 | 870.14 | 880.89 | 874.80 | 871.01 |
|  | BIC | 904.60 | 877.84 | 888.58 | 882.50 | 878.71 |

FUL: fulvestrant; PAL: palbociclib; AIC: Akaike information criterion; BIC: Bayesian information criterion; PFS: progression-free survival; OS: overall survival

Table S2 Best fitting and the value of the parameter

|  | Fitting | λ/μ | γ/σ |
| --- | --- | --- | --- |
| PFS |  |  |  |
| FUL | log-normal (μ,σ) | 1.60 | 1.00 |
| PAL+FUL | Gompertz (λ,γ) | 0.04 | 0.13 |
| OS |  |  |  |
| FUL | log-normal (μ,σ) | 3.38 | 0.99 |
| PAL+FUL | Log-logistic (λ,γ) | 0.03 | 0.56 |

FUL: fulvestrant; PAL: palbociclib; PFS: progression-free survival; OS: overall survival

# Detailed information of three clinical trials used in the study

Table S3 Detailed information of MONARCH 2

| **Study Name** | MONARCH 2  NCT02107703 |
| --- | --- |
| **Study Type** | A global, randomized (2:1), double-blind, placebo-controlled phase 3 study |
| **Arm** | Experimental: Abemaciclib + Fulvestrant |
|  | Placebo Comparator: Placebo + Fulvestrant |
| **inclusion criteria** | - Eligible women were ≥18 years old with any menopausal status (pre- or perimenopausal women received a gonadotropin-releasing hormone agonist) - Have a diagnosis of HR+, HER2- breast cancer and had an Eastern Cooperative Oncology Group performance status of 0 or 1 - Patients were required to have disease that progressed while receiving neoadjuvant or adjuvant ET, within 12 months from the end of adjuvant ET, or while receiving first-line ET for ABC |
| **exclusion criteria** | - Patients received more than 1 line of ET or any prior chemotherapy for ABC - Patients had prior treatment with fulvestrant, everolimus, or CDK4 and CDK6 inhibitors - The presence of visceral crisis - Evidence or history of central nervous system metastasis |
| **subsequent treatments** | Subsequent systemic therapies were received by 281 (63.0%) patients in abemaciclib arm and 180 (80.7%) in the placebo arm, including targeted agent therapy (bevacizumab, CDK4/6 inhibitor, everolimus), endocrine therapy (tamoxifen, fulvestrant, NSAI, exemestane) and chemotherapy (cyclophosphamide, vinorelbine, eribulin, anthracycline, capecitabine, taxane). |

Table S4 Detailed information of MONALEESA-3

| **Study Name** | MONALEESA-3  NCT02422615 |
| --- | --- |
| **Study Type** | A phase III, randomized (2:1), double-blind, placebo-controlled international study |
| **Arm** | Experimental: Ribociclib + fulvestrant |
|  | Placebo Comparator: Placebo + fulvestrant |
| **inclusion criteria** | - Postmenopausal women and men who were at least 18 years of age at trial entry with histologically and/or cytologically confirmed HR-positive/HER2 negative advanced (metastatic or locoregionally recurrent disease not amenable to curative treatment) breast cancer - The trial population included patients: (1) newly diagnosed (de novo), advanced breast cancer, (2) relapse＞12 months from completion of (neo)adjuvant endocrine therapy with no treatment for advanced or metastatic disease (criteria 1 and 2 referred to as treatment na¨ıve in the advanced setting hereafter), (3) relapse on or within 12 months from completion of (neo)adjuvant endocrine therapy with no treatment for advanced or metastatic disease (early relapse), (4) relapse＞12 months from completion of (neo)adjuvant therapy with subsequent progression after one line of endocrine therapy for advanced or metastatic disease, and (5) advanced or metastatic breast cancer at diagnosis that progressed after one line of endocrine therapy for advanced disease with no prior (neo)adjuvant treatment for early disease (criteria 3 to 5 referred to as received up to one line of endocrine therapy for advanced disease hereafter). Criteria 1 and 2 includes patients receiving treatment in the first-line setting; criteria 3 to 5 includes patients in the second-line setting or with an early relapse - Had adequate organ and bone marrow function and an Eastern Cooperative Oncology Group performance status of 0 or 1 |
| **exclusion criteria** | - Had received prior treatment with chemotherapy for advanced disease, fulvestrant, or a CDK4/6 inhibitor - Had inflammatory breast cancer, symptomatic visceral disease, or any disease burden that made the patient ineligible for endocrine therapy per investigator judgment - Had clinically significant cardiac arrhythmias and/or uncontrolled heart disease, including a QT interval corrected for heart rate according to Fridericia’s formula (QTcF) ＞450 ms |
| **subsequent treatments** | First subsequent antineoplastic medication type included chemotherapy alone, chemotherapy+ hormone therapy/other, hormone therapy alone, hormone therapy+ other and targeted therapy alone. All lines of subsequent antineoplastic medications mainly included chemotherapy (pyrimidine analogues, platinum compounds, anthracycline and taxane), hormone therapy (aromatase inhibitors and anti-estrogens) and kinase inhibitors (everolimus, palbociclib, abemaciclib, ribociclib and others). |

Table S5 Detailed information of PALOMA-3

| **Study Name** | PALOMA-3  NCT01942135 |
| --- | --- |
| **Study Type** | A prospective, randomised, double-blind, placebo-controlled phase 3 trial |
| **Arm** | Experimental: palbociclib + fulvestrant |
|  | Placebo Comparator: placebo + fulvestrant |
| **inclusion criteria** | - Had confirmed hormone receptor-positive, HER2-negative metastatic breast cancer - women aged 18 years or older of any menopausal status and with Eastern Cooperative Oncology Group performance status 0–1 - Disease relapse or progression had to occur after previous endocrine therapy (with an aromatase inhibitor if the patient was postmenopausal or with tamoxifen if premenopausal or perimenopausal) while on or within 1 month after treatment in the advanced setting, or while on or within 12 months of completion of adjuvant therapy irrespective of menopausal status. One previous line of chemotherapy in advanced disease was allowed. |
| **exclusion criteria** | - Had previously received any CDK inhibitor, fulvestrant, everolimus, or a PI3K/mTOR pathway inhibitor - Had extensive symptomatic visceral metastasis and were at risk of life-threatening complications in the short term - Had uncontrolled CNS metastases |
| **subsequent treatments** | The type of subsequent treatment was similar in the two trial groups, except for subsequent CDK4/6 inhibitor treatment. The chemotherapy mainly included eribulin, paclitaxel, capecitabine, doxorubicin, vinorelbine, gemcitabine, cyclophosphamide, carboplatin, and endocrine-based therapy mainly included exemestane and everolimus. Approximately 40% of the patients in each group received endocrine-based therapy as the immediate subsequent line of treatment. The median duration of subsequent therapy was similar in the two groups, and the median time to the receipt of chemotherapy was 17.6 months in the palbociclib–fulvestrant group, as compared with 8.8 months in the placebo fulvestrant group (hazard ratio, 0.58; 95% CI, 0.47 to 0.73; P<0.001). |

Table S6 Efficacy results of three clinical trials

| **study** | **Experimental group median PFS** | **Control group median PFS** | **HR** | **Experimental group median OS** | **Control group median OS** | **HR** | **≥grade 3 AEs with at least 5% incidence in two groups** |
| --- | --- | --- | --- | --- | --- | --- | --- |
| MONARCH 2 | 16.4 months | 9.3 months | 0.553  (0.449-0.681) | 46.7 months | 37.3 months | 0.757  (0,606-0.945) | neutropenia (29.7% vs 1.7%),  diarrhea (14.5% vs 0.4%),  leukopenia (11.1% vs 0%),  anemia (10% vs 1.3%) |
| MONALEESA-3 | 20.5  (18.5-23.5)  months | 12.8  (10.9-16.3) months | 0.593  (0.480-0.732) | not reached | 40 months | 0.72  (0.57-0.92) | neutropenia (57.1% vs 0.8%),  leukopenia (15.5% vs 0%),  hepatobiliary toxicity (13.7% vs 5.8%),  Infections (7.7% vs 3.7%) |
| PALOMA-3 | 9.5  (9.2-11.0)  month | 4.6  (3.5-5.6) months | 0.460  (0.36-0.59) | 34.9  (28.8-40.0) months | 28.0  (23.6-34.6) months | 0.81  (0.64-1.03) | neutropenia (69.6% vs 0%),  leukopenia (38.3% vs 0.6%),  Infections (5.2% vs 3.5%) |

PFS: progression-free survival; OS: overall survival; HR: hazard ratio; AE, advanced evens

Table S7 Comparable baseline population characteristics in three clinical trials

|  | Abemaciclib+Fulvestrant | Placebo+Fulvestrant | Ribociclib+Fulvestrant | Placebo+Fulvestrant | Palbociclib+Fulvestrant | Placebo+Fulvestrant |
| --- | --- | --- | --- | --- | --- | --- |
|  | n=446 | n=223 | n=484 | n=242 | n=347 | n=174 |
| Age,years,median (range) | 59 (32-91) | 62 (32-87) | 63 (31-89) | 63 (34-86) | 57 (30-88) | 56 (33-88) |
| ECOG performance status |  |  |  |  |  |  |
| 0 | 264 (59.2%) | 136 (61.0%) | 310 (64.0%) | 158 (65.3%) | 206 (59%) | 116 (67%) |
| 1 | 176 (39.5%) | 87 (39.0%) | 173 (35.7%) | 83 (34.3%) | 141 (41%) | 58 (33%) |
| Race |  |  |  |  |  |  |
| White | 237 (53.1%) | 136 (61.0%) | 406 (83.9%) | 213 (88.0%) | 252 (73%) | 133 (76%) |
| Asian | 149 (33.4%) | 65 (29.1%) | 45 (9.3%) | 18 (7.4%) | 74 (21%) | 31 (18%) |
| Black | 29 (6.5%) | 13 (5.8%) | 3 (0.6%) | 2 (0.8%) | 21 (6%) | 10 (6%) |
| Others |  |  | 10 (2.1%) | 3 (1.2%) |  |  |
| Menopausal status |  |  |  |  |  |  |
| Pre- or perimenopause | 72 (16.1%) | 42 (18.8%) | / | / | 72 (21%) | 36 (21%) |
| Postmenopause | 371 (83.2%) | 180 (80.7%) | / | / | 275 (79%) | 138 (79%) |
| Metastatic site |  |  |  |  |  |  |
| Visceral | 245 (54.9%) | 128(57.4%) | 293 (60.5%) | 146 (60.3%) | / | / |
| Bone only | 123 (27.6%) | 57 (25.6%) | 103 (21.3%) | 51 (21.1%) | / | / |
| Others | 75 (16.8%) | 38 (17.0%) | / | / | / | / |

ECOG=Eastern Cooperative Oncology Group
